# A Miocene pyrgodesmid millipede (Polydesmida: Pyrgodesmidae) from Mexico

**DOI:** 10.7717/peerj.10574

**Published:** 2021-01-25

**Authors:** Francisco Riquelme, Miguel Hernández-Patricio, Michelle Álvarez-Rodríguez

**Affiliations:** Laboratorio de Sistemática Molecular. Escuela de Estudios Superiores del Jicarero, Universidad Autónoma del Estado de Morelos, Jojutla, Morelos, Mexico

**Keywords:** Miocene, Mexico, Diplopoda, Pyrgodesmidae, New species

## Abstract

A new fossil species of pyrgodesmid millipede (Polydesmida: Pyrgodesmidae) placed in the genus *Myrmecodesmus* Silvestri, 1910 is described. The type materials are two amber inclusions, male and female specimens that come from Miocene strata in Chiapas, Mexico. *Myrmecodesmus antiquus* sp. nov. has collum with 10 dorsal tubercles; without porosteles or ozopores; legs of the rings 2–9 with a short projection on the prefemur in both the female and male. *Myrmecodesmus antiquus* sp. nov is the first fossil record of the genus *Myrmecodesmus*. This is a New World taxon that belongs to the pantropical family Pyrgodesmidae. Thus, *Myrmecodesmus antiquus* sp. nov expands the range of the genus to the Miocene tropics in Middle America.

## Introduction

The polydesmid millipedes of the family Pyrgodesmidae currently show a Pantropical distribution (Enghoff et al., 2015). However, the fossil record is limited to amber inclusions from Miocene deposits of the Dominican Republic and Mexico (Shear, 1981; Santiago-Blay & Poinar, 1992; Riquelme & Hernández-Patricio, 2018). Fossil specimens of Dominican amber have been assigned to the genera *Docodesmus* Cook, 1896a, *Iomus* Cook, 1911, *Lophodesmus* Pocock, 1894 *and Psochodesmus* Cook, 1896b (Shear, 1981; Santiago-Blay & Poinar, 1992), and the only fossil specimen described at the species level that is known so far is *Docodesmus brodzinskyi* Shear, 1981. Another fossil pyrgodesmid was found in Chiapas amber, Mexico, a female specimen identified as CPAL.117 by Riquelme & Hernández-Patricio (2018), which was initially included as a new member of the genus *Myrmecodesmus* within Pyrgodesmidae. In the present contribution, based on the female CPAL.117 plus another male specimen identified as CPAL.132, a new species of the genus *Myrmecodesmus* is now described. Below are descriptions, illustrations, and a discussion of related taxa.

## Geological setting

The fossil specimens CPAL.117 and CPAL.132 come from the lignite-sandstones beds exposed in a site called Los Pocitos in the town of Simojovel, located approximately 122 km by road from the city of Tuxtla, Chiapas, southwestern Mexico. The amber-bearing beds of Simojovel are generally assigned to the Mazantic and Balumtum strata from early to mid-Miocene (Perriliat, Vega & Coutiño, 2010; Durán-Ruíz et al., 2013; Riquelme et al., 2013). Another outcrop exposed near Los Pocitos in Simojovel that contains fossil amber, is preliminarily considered the upper portion of the La Quinta strata in the late Oligocene (Graham, 1999). Here, a marine sedimentary environment is predominantly observed. The stratigraphic section and lithology of a typical amber outcrop in Chiapas are presented in Durán-Ruíz et al. (2013). It is indicated there that a portion of the marine sandstones of the La Quinta is located between the boundaries of the early Miocene and the late Oligocene. However, the complete geology of all amber deposit in Chiapas is an unresolved issue and the stratigraphic record of amber outcrops must be carefully considered (Durán-Ruíz et al., 2013; Riquelme et al., 2013). We consistently noted in the field that most of the fossil inclusions in Simojovel, Totolapa and Estrella de Belén in the Chiapas Highlands come from lignite-sandstones beds which belong to the Mazantic and Balumtum strata from early to mid-Miocene (Durán-Ruíz et al., 2013; Riquelme et al., 2013, 2014a, 2014b). Simojovel, Totolapa, and Estrella de Belén are considered the type localities of a Conservation Lagerstätte with a remarkable abundance of amber inclusions, predominantly terrestrial arthropods and plants (Riquelme et al., 2013, 2014b). Here the sedimentary record is strongly associated with a lowland-fluvial environment close to the coastal plain (Graham, 1999; Langenheim, 2003; Perriliat, Vega & Coutiño, 2010; Riquelme et al., 2013); and paleobiota resembles those found in current humid tropics (Riquelme & Hernández-Patricio, 2018). Chiapas amber has chemical signatures that match with the extant resins of the genus *Hymenaea* (sensu Langenheim, 1966), which are also currently distributed in the tropics (Langenheim, 2003; Riquelme et al., 2014b)

## Materials and Methods

The fossil specimens treated in this study are listed as CPAL.117 and CPAL.132, housed at the Colección de Paleontología, Universidad Autónoma del Estado de Morelos (CPAL-UAEM), located in Cuernavaca, Morelos, Mexico. Anatomical terminology follows Hoffman (1976) and Koch (2015), and nomenclature follows Shear (2011). Preparation of material and methods used here are presented in Riquelme et al. (2013). Microphotographs were acquired using multiple image-stacking (Z ≥ 45) in a Carl Zeiss microscope, and schematic drawings were hand traced by electronic pen using a stereomicroscope and Corel Draw X7 for graphic processing. Anatomical measurements are presented in millimeters and were collected using the open-source program tpsDig V. 2.17 (Rohlf, 2013).

The electronic version of this article in Portable Document Format will represent a published work according to the International Commission on Zoological Nomenclature (ICZN), and hence the new names contained in the electronic version are effectively published under that Code from the electronic edition alone. This published work and the nomenclatural acts it contains have been registered in ZooBank, the online registration system for the ICZN. The ZooBank Life Science Identifiers can be resolved and the associated information viewed through any standard web browser by appending the LSID to the prefix http://zoobank.org/. The LSID for this publication is: urn:lsid:zoobank.org:pub:95759D56-205C-4FD2-A01B-0CE3F9621E0E. The online version of this work is archived and available from the following digital repositories: PeerJ, PubMed Central and CLOCKSS.

## Results

### Systematic paleontology

Order Polydesmida Pocock, 1887

Suborder Polydesmidea Pocock, 1887

Infraorder Oniscodesmoides Simonsen, 1990

Superfamily Pyrgodesmoidea Silvestri, 1896

Family Pyrgodesmidae Silvestri, 1896

Genus *Myrmecodesmus* Silvestri, 1910

*Myrmecodesmus antiquus* sp. nov. Riquelme and Hernández-Patricio.

Figs. 1–5.

**Figure 1:**
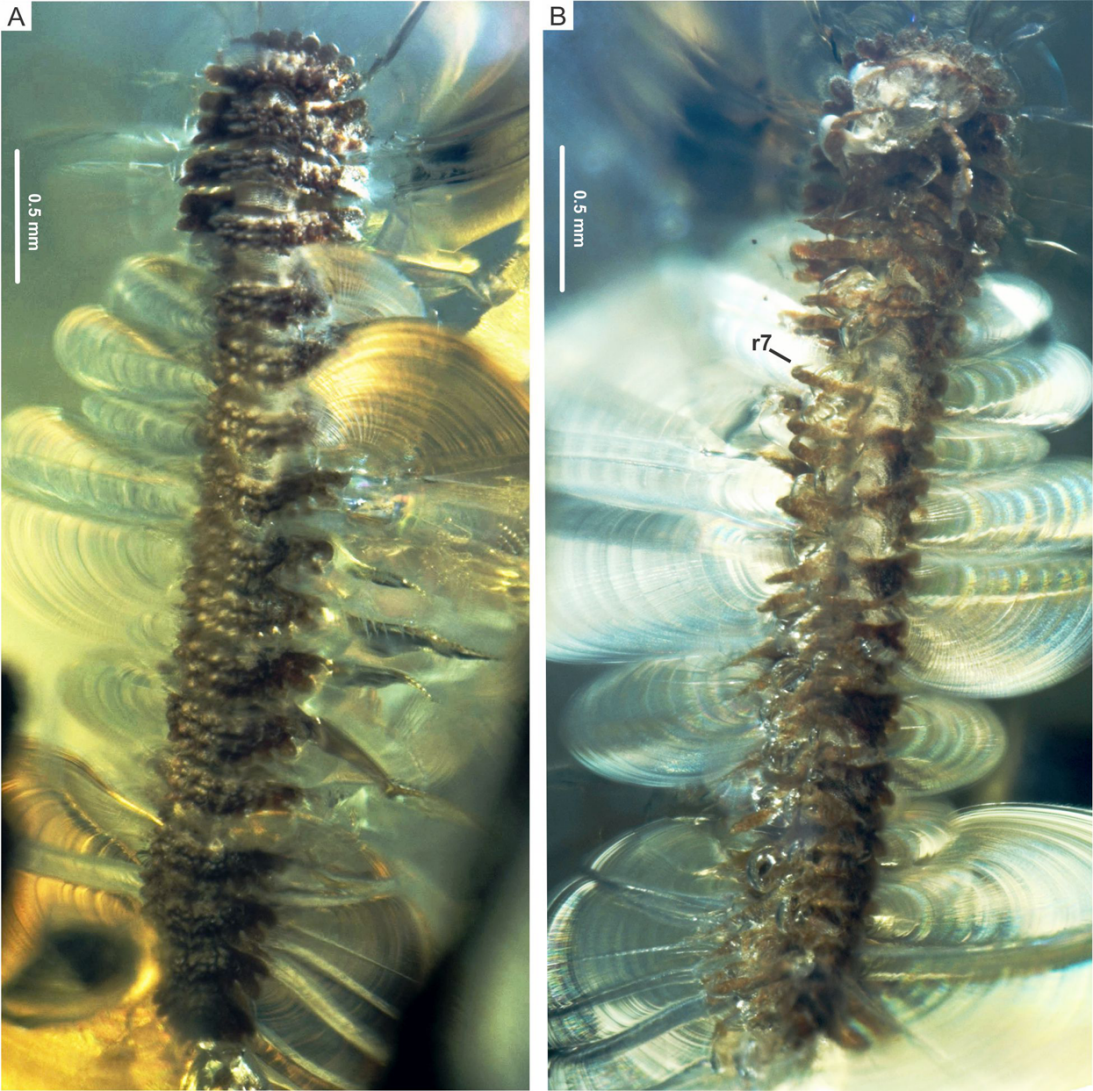
*Myrmecodesmus antiquus* sp. nov. Holotype CPAL.132, amber inclusion, complete fossil specimen, male adult. (A) Dorsal view. (B) Ventral view, showing ring 7. Abbreviation: r, body ring.

**Figure 2:**
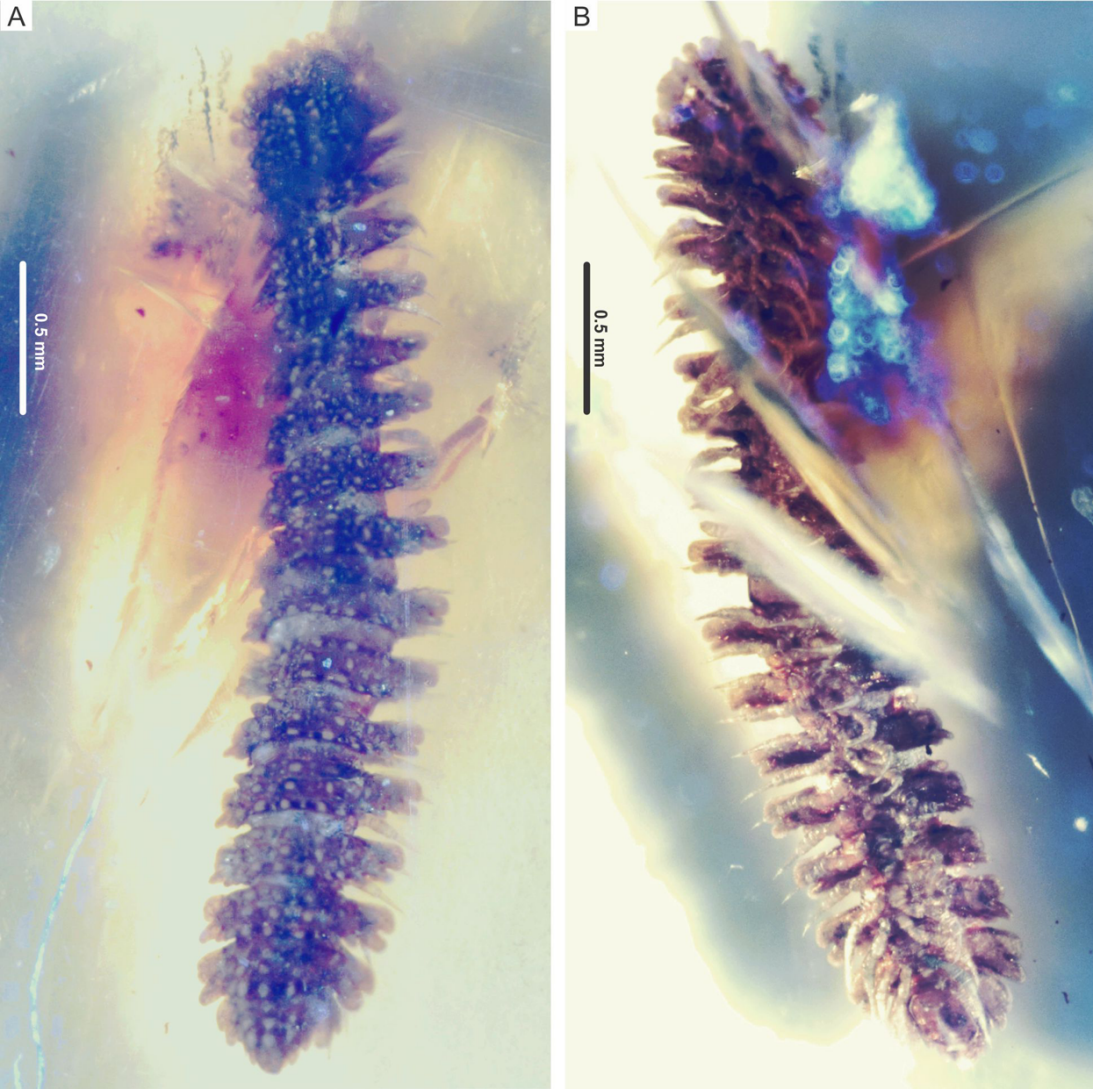
*Myrmecodesmus antiquus* sp. nov. Paratype CPAL.117, amber inclusion, complete fossil specimen, female adult. (A) Dorsal view. (B) Ventral view.

**Figure 3:**
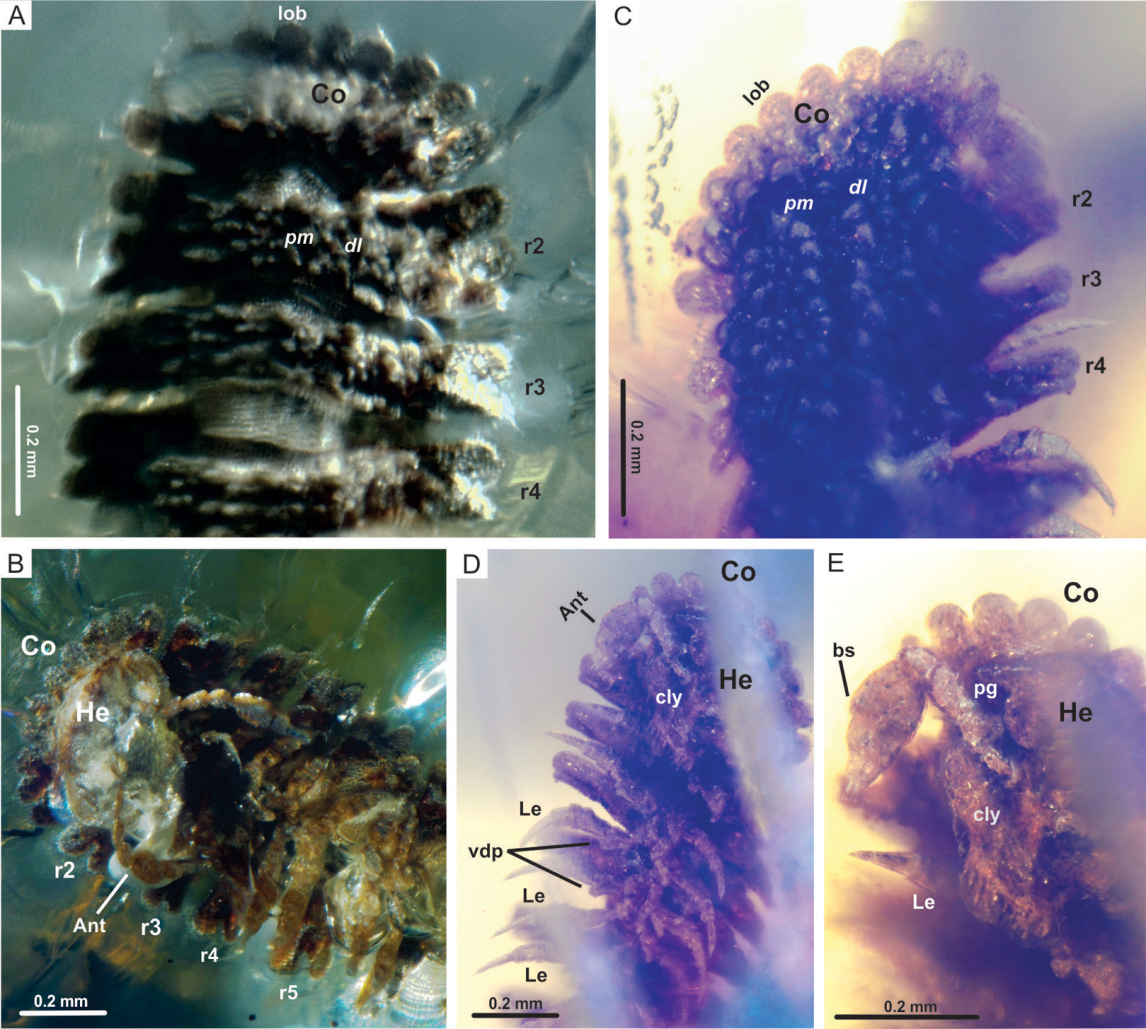
*Myrmecodesmus antiquus* sp. nov. (A) CPAL.132, male, dorsal view of anterior end, showing collum and rings 2–4. (B) CPAL.132, ventral view of anterior end, showing head, antenna, collum lobes, and rings 2–5. (C) CPAL.117, female, dorsal view of anterior end, showing collum and rings 2–4. In both specimens: collum with 10 lobes and rings 2–4 with two paramedian and two dorsolateral tubercle rows. (D) CPAL.117, ventral view of anterior end, showing head, antenna, rings 2–6, and associated legs with ventrodistal projection on the prefemur. (E) CPAL.117, closer view at the head and antenna with bacilliform sensilla. Abbreviations: Ant, antenna; bs, bacilliform sensilla; cly, clypeus; Co, collum; *dl*, dorsolateral tubercle; He, head; Le, leg; lob, lobe; *pm*, paramedian tubercle; pg, postantennal groove; r, body ring; vdp, ventrodistal projection.

**Figure 4:**
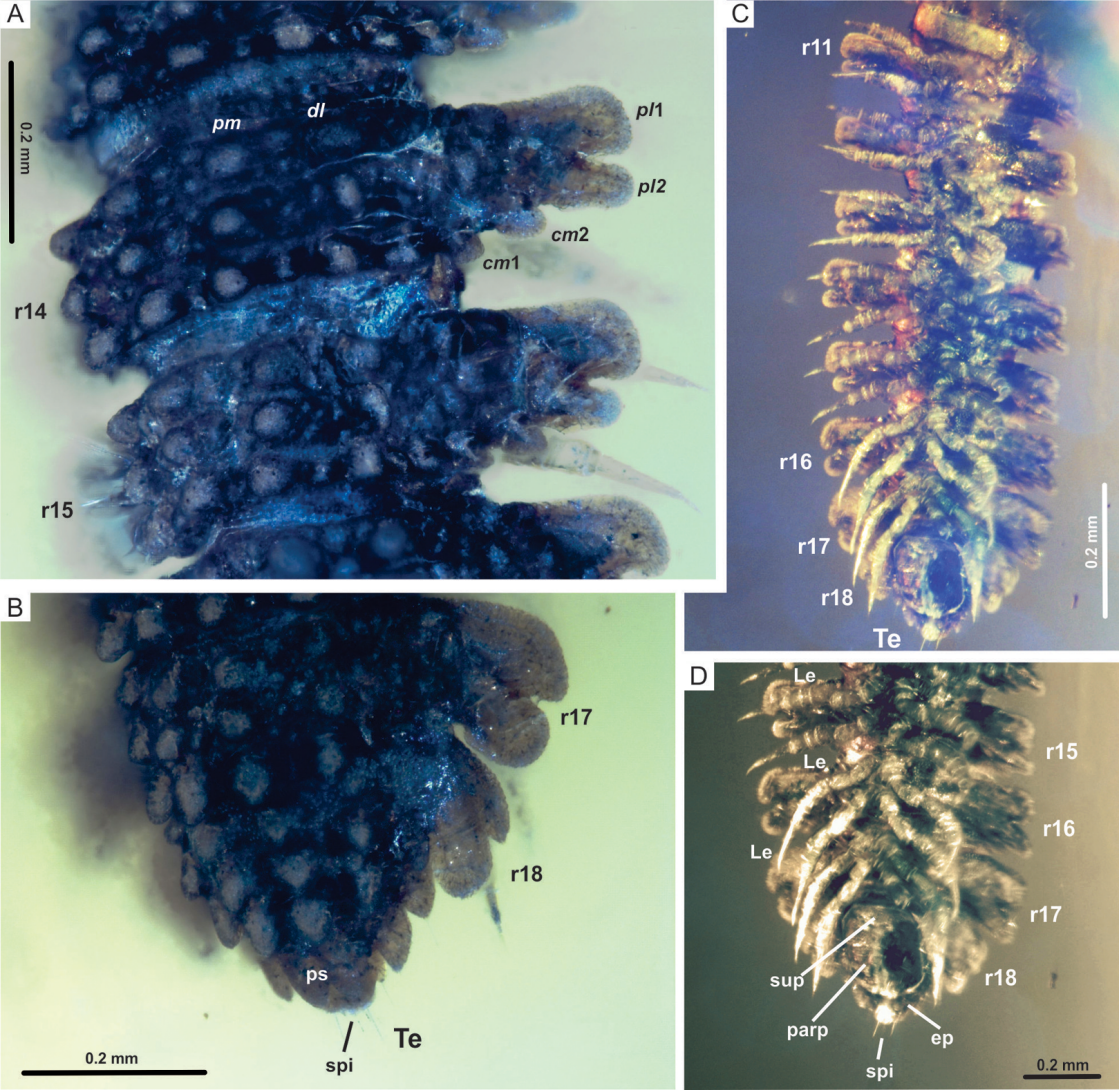
*Myrmecodesmus antiquus* sp. nov. (A) CPAL.117, dorsal view of rings 14 and 15, showing paramedian and dorsolateral tubercles, and paranota with two caudomarginal and two paranotal lobes. (B) CPAL.117, dorsolateral view of posterior end, showing rings 16–18, and telson. (C) CPAL.117, ventral view of midbody rings and posterior end, showing rings 11–18, and telson. (D) CPAL.117, closer view at the posterior end, ventral view, showing subanal plate, paraprocts, epiproct, and spinnerets. Abbreviations: *cm*, caudomarginal lobe; *dl*, dorsolateral tubercle; ep, epiproct; Le, leg; parp, paraprocts; *pl*, paranotal lobe; *pm*, paramedian tubercle; ps, preanal sclerite; r, body ring; spi, spinneret; sup, subanal plate; Te, telson.

**Figure 5:**
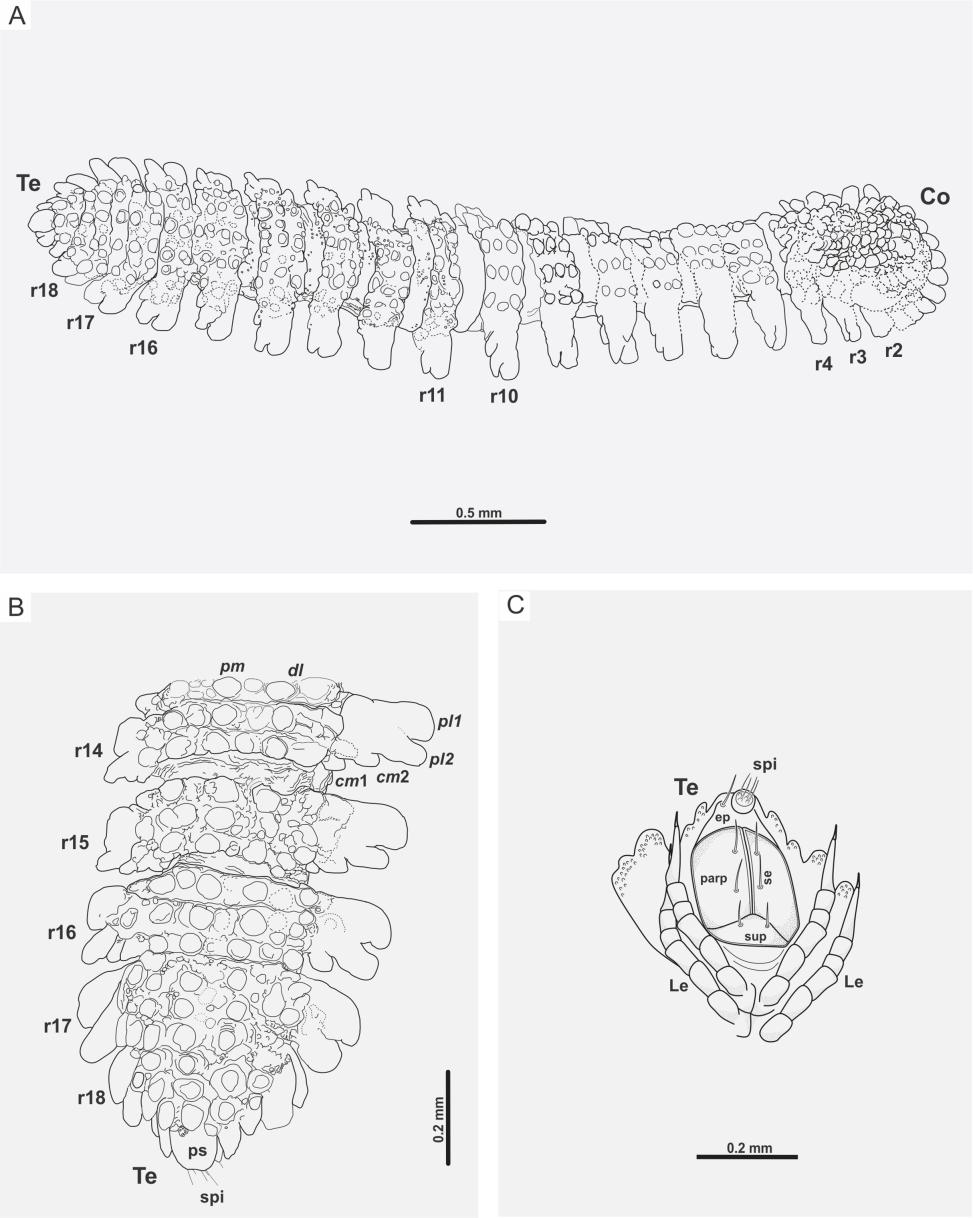
*Myrmecodesmus antiquus* sp. nov. (A) CPAL.117, line drawing of the complete fossil specimen, dorsal view, showing collum, trunk, and telson. (B) CPAL.117, schematic reconstruction of posterior end, dorsal view, showing rings 14–18, and telson. (C) CPAL.117, schematic reconstruction of telson, ventral view, showing subanal plate, paraprocts, epiproct, spinnerets, setae, and associated legs in posterior end. Abbreviations: *cm*, caudomarginal lobe; Co, collum; *dl*, dorsolateral tubercle; ep, epiproct; Le, leg; parp, paraprocts; *pl*, paranotal lobe; *pm*, paramedian tubercle; ps, preanal sclerite; r, body ring; se, setae; spi, spinneret; sup, subanal plate; Te, telson.

ZooBank LSID: urn:lsid:zoobank.org:act:EA1B5DD9-14AC-46DB-9D00-3D699F84C296

**Etymology**. From the Latin word “antiquus” (m.), which means “ancient”. It alludes to the fossil condition of the specimens.

**Type material**. Holotype CPAL.132 (Fig. 1), amber inclusion, entire specimen, male adult with 19 rings, housed at the CPAL-UAEM (2019). Paratype CPAL.117 (Fig. 2), amber inclusion, entire specimen, female adult with 19 rings, housed at the CPAL-UAEM (2017).

**Locality and Horizon**. Los Pocitos site, Simojovel, Chiapas, Mexico, latitude 17° 08′ 32″ N, longitude 92° 43′ 27″ W. The amber-bearing rocks belong to Mazantic shale and Balumtum sandstone strata dated as early-middle Miocene, ca. 23–15 Ma (Poinar, 1992; Perriliat, Vega & Coutiño, 2010; Riquelme et al., 2013).

**Diagnosis.** With traits of the genus *Myrmecodesmus* sensu Shear (1977), plus the following combination of diagnostic characters: collum with 10 dorsal tubercles; without porosteles or ozopores; legs of the rings 2–9 with a short projection on the prefemur in both female and male.

**Description.** Color preserved in amber, brownish-gray from the dorsal view, and pale gray in ventral view. Holotype CPAL.132, male, complete specimen (Fig. 1). Paratype CPAL.117, female, complete specimen (Fig. 2). Measurements (in mm): CPAL.132: head plus 19 rings, length 3.7, collum width 0.6, metazonite width 0.72, prozonite width 3.0. CPAL.117: head plus 19 rings, length 3.3, collum width 0.57, metazonite 0.68, prozonite width 0.28 (Figs. 1 and 2).

Head: vertex and frons roughened, partially sunk, slightly granular surface, clypeus also granular, with 10–12 setae (Figs. 1B, 2B, 3B and 3D)

Antenna: consisting of 7 antennomeres; postantennal groove deep; antennal sockets separated by ca 1× the socket diameter; antennae short, stout and clavate. The antennomere are widest in the female CPAL.117. Antennomere 5 widest. Antennomere relative widths 5 > 6 > 7 > (4 = 3 =2 = 1), relative lengths (3 = 5) >2 > 6 > 4 > 7 > 1 and four apical, long, and slender sensory cones. Distinctly tight group of bacilliform sensilla on the apical, retrolateral edge of antennomere 5 (Figs. 3B, 3D and 3E).

Trunk: collum flabellate covering head in dorsal view; anterior margin divided into 10 equal rounded lobes separated and parallel to ground; dorsal surface domed with 2 transverse rows of tubercles, posterior row with 4 large tubercles, medial row with 6 medium tubercles, the rest of the collum surface with small tubercles (Figs. 3A and 3C). Midbody metazonite surface with 4 longitudinal rows of 2 paramedian (*pm*) and 2 dorsolateral (*dl*) tubercles, composed of 3 large tubercles that are basally fused; a group of 4 or 6 small tubercles intercalate between *dl* and *pm* tubercles, and 2 rows of 5 or 6 small tubercles middorsally, a variable number of medium and small tubercles between *dl* tubercles and paranota; collum, tergites and metazonites lacking setae. Prozonites slightly granular, some margins with tiny tubercle-like cuticular outgrowths (Figs. 4A, 4B and 5A). Paranota medium-sized, arising low on body, slightly directed anteriorly, declined, anterior margin straight, undivided, pitched slightly so anterior margin is lower than posterior margin. Ring 2 paranotum expanded anterodistally, lateral margin weakly divided with typical 3 rounded lateral paranota lobes (*pl*); *pl*1, *pl*2 and *pl*3 are sub-equal, lacking caudomarginal (*cm*) and anteriormarginal (*am*) lobes (Figs. 3A and 3C). Ring widths 3–16 about equal, gradually decreasing on rings 17–18 (Figs.1, 2, 3, 4 and 5A). Paranota of rings 3–18, with anterior margin straight, undivided; 2 strong *pl*; posterior margin with two caudomarginal lobes, *cm*1 shorter than *cm*2. Paranota of rings 17–18 directed posterolaterally, ring 17 with one *cm*, and ring 18 without *cm*. Ozopores not visible, without porosteles (Figs.1A, 2B, 4A, 4B, 5A and 5B).

Legs: subequal, short and slender, hidden by paranota in dorsal view; relative podomere lengths tarsus>femur>prefemur>(postfemur = tibia)>coxa and with a long claw. Legs with modified prefemur in rings 2–9, with a short ventrodistal projection protruding. Spiracles not evident (Figs. 3B, 3D, 4C and 4D).

Telson: preanal sclerite with 3+3 lateral lobes and 2 small dorsal tubercles; epiproct not hidden under 18th segment, short, bluntly rounded, with 4 strong spinnerets in square array below apex; anal valves each with 2 setae near the mesal margin; subanal plate rounded-triangular with 2 setae near the apex. Sternites slightly roughened, not setose, wider than long; coxae nearly contiguous, with a transverse impression slightly deeper than longitudinal (Figs. 4C, 4D, 5B and 5C).

**Remarks.** Gonopods are not visible in the male CPAL.132 and the epigyne and cyphopods are also not distinguishable in the female CPAL.117, as a consequence of the state of conservation of the bodies and because they are partially covered with cloudy amber. However, CPAL.132 and CPAL.117 are interpreted as male and female adults, respectably, due to the number of rings =19 (extant species of *Myrmecodesmus* may have either 19 or 20 rings as adults), the paranota and metazonite tubercles strongly differentiated, as well as the number of legs in ring 7 of the male (Figs. 1B, 2B and 3B–3D).

## Discussion

Unresolved taxonomic issues persist in the genus *Myrmecodesmus* (Shear, 1973; Shelley, 2002; Recuero, 2014). A large number of the currently valid species show partial taxonomic descriptions and there is no key or consensus on the diagnostic characters in the genus. This leads to certain uncertainties in the species diagnosis. The synapomorphies of the group also seems ambiguous. At present, it is an unsolved problem in Diplopoda taxonomy (Shear, 1973, 1977; Hoffman, 1980; Hoffman et al., 1996; Adis, 2002; Golovatch & VandenSpiegel, 2014; Recuero, 2014). *Myrmecodesmus* is a diverse genus with some of the highest species numbers that exist within the family Pyrgodesmidae (Golovatch, 1999; Golovatch & Adis, 2004; Shelley, 2004; Golovatch et al., 2016). Table 1 shows an updated list of the valid species that belong to *Myrmecodesmus*, according to the literature reviewed to date. It is a group that currently comprises 36 described extant species (Shear, 1977; Reddell, 1981; Golovatch, 1999; Hoffman, 1999; Shelley, 2004; Recuero, 2014). Accordingly, *M. antiquus* sp. nov. is the only fossil species described in the genus so far.

**Table 1:** The current list of species in the genus *Myrmecodesmus* Silvestri, 1910 (Diplopoda: Polydesmida: Pyrgodesmidae), including the fossil *Myrmecodesmus antiquus* sp. nov.

	Species	Distribuction	Source	
1	*Myrmecodesmus aconus* Shear, 1973	Mexico	Shear (1977)	
2	*Myrmecodesmus adisi* Hoffman, 1985	Brazil	Golovatch (1999)	
3	*Myrmecodesmus amarus* Causey, 1971	Mexico	Shear (1977)	
4	*Myrmecodesmus amplus* Causey, 1973	Mexico	Shear (1977)	
5	*Myrmecodesmus analogous* Causey, 1971	Mexico	Shear (1977)	
6	†*Myrmecodesmus antiquus* Riquelme & Hernández-Patricio, 2021	Miocene, Mexico	Riquelme et al. (2021)	
7	*Myrmecodesmus atopus* Chamberlin, 1943	Mexico	Shear (1977)	
8	*Myrmecodesmus brevis* Shear, 1977	Mexico	Shear (1977)	
9	*Myrmecodesmus chamberlini* Shear, 1977	Mexico	Shear (1977)	
10	*Myrmecodesmus chipinqueus* Chamberlin, 1943	Mexico	Shear (1977)	
11	*Myrmecodesmus clarus* Chamberlin, 1942	Mexico	Shear (1977)	
12	*Myrmecodesmus cornutus* Shear, 1973	Mexico	Shear (1977)	
13	*Myrmecodesmus digitatus* Loomis, 1959	USA	Hoffman (1999)	
14	*Myrmecodesmus duodecimlobatus* Golovatch, 1996	Brazil	Golovatch (1999)	
15	*Myrmecodesmus egenus* Causey, 1971	Mexico	Shear (1977)	
16	*Myrmecodesmus errabundus* Shear, 1973	Mexico	Shear (1977)	
17	*Myrmecodesmus fissus* Causey, 1977	Mexico	Reddell (1981)	
18	*Myrmecodesmus formicarius* Silvestri, 1910	Mexico	Shelley (2004)	
19	*Myrmecodesmus fractus* Chamberlin, 1943	Mexico	Shear (1977)	
20	*Myrmecodesmus fuscus* Causey, 1977	Mexico	Reddell (1981)	
21	*Myrmecodesmus gelidus* Causey, 1971	Mexico	Hoffman (1999)	
22	*Myrmecodesmus hastatus* Schubart, 1945	Brazil, Peru, Argentina, Martinique, Lesser Antilles	Golovatch & Adis (2004)	
23	*Myrmecodesmus ilymoides* Shear, 1973	Mexico	Shear (1977)	
24	*Myrmecodesmus inornatus* Shear, 1977	Mexico	Shear (1977)	
25	*Myrmecodesmus margo* Causey, 1977	Mexico	Causey (1977)	
26	*Myrmecodesmus minusculus* Golovatch, 1996	Brazil	Golovatch (1999)	
27	*Myrmecodesmus modestus* Silvestri, 1911	Mexico	Silvestri (1911)	
28	*Myrmecodesmus monasticus* Causey, 1971	Mexico	Shear (1977)	
29	*Myrmecodesmus morelus* Chamberlin, 1943	Mexico	Shear (1977)	
30	*Myrmecodesmus mundus* Chamberlin, 1943	Mexico	Shear (1977)	
31	*Myrmecodesmus obscurus* Causey, 1971	Mexico	Shear (1977)	
32	*Myrmecodesmus orizaba* Chamberlin, 1941	Mexico	Shear (1977)	
33	*Myrmecodesmus potosinus* Chamberlin, 1943	Mexico	Shear (1977)	
34	*Myrmecodesmus reddelli* Shelley, 2004	USA	Shelley (2004)	
35	*Myrmecodesmus sabinus* Chamberlin, 1942	Mexico	Shear (1977)	
36	*Myrmecodesmus sheari* Recuero, 2014	Mexico	Recuero (2014)	
37	*Myrmecodesmus unicorn* Shear, 1977	Belize	Shear (1977)	

Among the Polydesmida, the family Pyrgodesmidae shows a Pantropical distribution, which includes southern USA, Mexico, Central America, the Antilles, South America, South Europe, Africa, Asia, India and Oceania (Hoffman, 1980, 1999; Golovatch, 1996; Shelley & Golovatch, 2001; Golovatch & Kime, 2009; Jorgensen & Sierwald, 2010; Mesibov, 2012; Enghoff et al., 2015). The living members of the Pyrgodesmidae count 371 nominal species included in 170 genera, most of them monotypes (Jorgensen & Sierwald, 2010; Enghoff et al., 2015). For its part, the genus *Myrmecodesmus* is an exclusively New World taxon, and the extant species of *Myrmecodesmus* are distributed in USA, Mexico, Belize, the Antilles, Peru, Brazil and Argentina (Golovatch, 1999; Hoffman, 1999; Bueno-Villegas, Sierwald & Bond, 2004; Golovatch & Adis, 2004; Bergholz, Adis & Golovatch, 2004; Shelley, 2004; Golovatch et al., 2016). Most species have been described from Mexico, and the genus has a current distribution in the Nearctic and Neotropical regions (Table 1). Originally, *Myrmecodesmus* was erected by Silvestri, 1910 to group some related morphotypes found in Veracruz, southern Mexico (Recuero, 2014). Thus, the occurrence of *M. antiquus* sp. nov. expands the range of the genus to the Miocene tropics in Middle America.

## Additional Information and Declarations

### Data Availability

The following information was supplied regarding data availability: The material is deposited at the Colección de Paleontología, Universidad Autónoma del Estado de Morelos (CPAL-UAEM): Code numbers: CPAL.117, CPAL.132. Raw images are available in Figs. 1–5.

### New Species Registration

The following information was supplied regarding the registration of a newly described species: Publication LSID: urn:lsid:zoobank.org:pub:95759D56-205C-4FD2-A01B-0CE3F9621E0E. *Myrmecodesmus antiquus* LSID: urn:lsid:zoobank.org:act:EA1B5DD9-14AC-46DB-9D00-3D699F84C296.
